# Comparative detection of respiratory pathogens in pediatric and adult patients using a multiplex panel

**DOI:** 10.1097/MD.0000000000049737

**Published:** 2026-07-10

**Authors:** Young Jin Kim, Woong Sik Jang, Seoyeon Park, Imseok Kang, Soo Young Yoon, Chae Seung Lim, Min-Chul Cho

**Affiliations:** a Department of Laboratory Medicine, Kyung Hee University Hospital, Kyung Hee University School of Medicine, Seoul, Republic of Korea; b Department of Emergency Medicine, Korea University Guro Hospital, Korea University College of Medicine, Seoul, Republic of Korea; c Department of Laboratory Medicine, Korea University Guro Hospital, Korea University College of Medicine, Seoul, Republic of Korea; d Department of Laboratory Medicine, Seegene Medical Foundation, Seoul, Republic of Korea.

**Keywords:** adults, bacterial pathogens, co-detection, multiplex panel testing, pediatric patients, respiratory tract infections, viral pathogens

## Abstract

The coronavirus disease 2019 pandemic has further complicated the epidemiology of respiratory tract infections by altering the circulation patterns of respiratory viruses and exacerbating diagnostic challenges. This study aimed to compare respiratory pathogen profiles between pediatric and adult populations during a period of increased respiratory pathogen activity in 2024, with a focus on co-detection patterns and pathogen identification. We analyzed data from 618 patients who visited Korea University Guro Hospital with acute respiratory symptoms between August and September 2024. Specimens were tested using multiplex polymerase chain reaction assays targeting both viral and bacterial pathogens. Demographic data were extracted from electronic medical records, and statistical analyses were performed to compare the pathogen profiles and co-detection rates between pediatric and adult groups. A total of 245 pediatric patients and 373 adult patients were included. Enterovirus/rhinovirus was the most common finding in pediatric patients (21.0%), whereas severe acute respiratory syndrome coronavirus 2 was most frequently detected in adults (32.9%). Co-detection of multiple pathogens was observed in 35 adult and 39 pediatric cases. In pediatric patients, co-detection of *Mycoplasma pneumoniae* and enterovirus/rhinovirus was observed in 5.7% of cases. The prevalence of *M pneumoniae* was higher in the pediatric cohort, with a male predominance. Severe acute respiratory syndrome coronavirus 2 was detected in 60 adult cases and 7 pediatric cases. These findings highlight notable age-related differences in the pathogen profiles of acute respiratory infections. This study provides epidemiological data regarding respiratory pathogen detection and co-detection patterns across age groups.

## 1. Introduction

Respiratory tract infections continue to pose a significant global health burden, with a wide range of viral, bacterial, and atypical pathogens contributing to seasonal outbreaks and epidemics.^[1–3]^ In recent years, the landscape of respiratory infections has become increasingly complex due to the emergence and reemergence of diverse pathogens. This complexity was further accelerated by the coronavirus disease 2019 (COVID-19) pandemic, which disrupted the typical circulation patterns of respiratory viruses worldwide.

Following the pandemic, particularly during 2022 to 2023, significant shifts in the circulation patterns of respiratory viruses were observed. As non-pharmaceutical interventions aimed at limiting severe acute respiratory syndrome coronavirus 2 (SARS-CoV-2) transmission were relaxed, a resurgence of multiple respiratory pathogens, such as influenza viruses, respiratory syncytial virus (RSV), *Mycoplasma pneumoniae*, and even *Bordetella pertussis*, was observed across various regions.^[2,4,5]^ Some viruses, such as RSV, human metapneumovirus, and parainfluenza viruses, showed atypical seasonality, with peaks occurring at times that differed from historical patterns.^[6]^ Furthermore, an increase in hospitalizations was observed in the spring of 2023 due to all respiratory viruses except human coronaviruses.^[6,7]^ In particular, pediatric RSV infections, which had decreased during the pandemic, surged after the decline of COVID-19, accompanied by an increase in bacterial pneumonia cases.^[8]^ These changes are likely attributable to the easing of COVID-19-related preventive measures and the return to regular social interactions.

These epidemiological shifts have also contributed to atypical clinical presentations, making symptom-based diagnosis increasingly unreliable.^[3,9]^ The limitations of symptom-based diagnosis have become more evident, particularly in the context of overlapping presentations and rising cases of co-infection. Consequently, accurate pathogen identification through laboratory-based diagnostics has become essential for guiding appropriate treatment and infection-control strategies.^[10,11]^

Given this complex background, the clinical significance of respiratory co-infections has gained increasing attention. Co-infections involving viral and/or bacterial pathogens may influence disease severity, prolong illness duration, complicate clinical diagnosis, and lead to inappropriate or delayed treatment. However, real-world data on the patterns and implications of co-infections across different age groups remain limited.

This study was conducted during a period of increased respiratory pathogen activity between August and September 2024, when multiple bacterial and viral pathogens, including SARS-CoV-2, were concurrently circulating. Comprehensive diagnostic testing was performed to identify the causative agents of acute respiratory infections in both adult and pediatric patients. The primary aim of this study was to compare respiratory pathogen profiles between adult and pediatric patients and to assess the patterns, characteristics, and proportions of co-detected respiratory pathogens in each age group. By identifying the most commonly observed combinations of pathogens in each age group, we sought to provide foundational epidemiological data that can support clinical decision-making.

## 2. Materials and methods

### 2.1. Study subjects and data collection

This retrospective study included patients who visited Korea University Guro Hospital between August 1, 2024, and September 30, 2024, with symptoms suggestive of acute respiratory tract infection, such as fever, chills, cough, sputum, or sore throat, and who underwent respiratory multiplex polymerase chain reaction (PCR) testing as part of routine clinical care. Both outpatients and inpatients were eligible for inclusion.

Patients were excluded if they underwent multiplex PCR testing for screening or infection-control purposes without symptoms suggestive of acute respiratory infection, if the PCR result was invalid or inconclusive, if essential demographic or specimen-related information was unavailable, or if the specimen was collected outside the study period. If multiple respiratory specimens were collected from the same patient during the study period, only the first valid test result was included in the analysis.

Residual extracted nucleic acid samples from these tests were used. All specimens were stored in a −70°C freezer until additional analyses were performed.

Demographic data were collected from electronic medical records. Demographic characteristics included age and gender. This study was approved by the Institutional Review Board of Korea University Guro Hospital (IRB No. 2024GR0127), which waived the requirement for informed consent.

### 2.2. Specimen collection and nucleic acid extraction

Upper respiratory specimens, including nasopharyngeal swabs (NPSs) and sputum samples, were collected. NPSs were obtained using flocked swabs and transported in 2.0 mL of universal transport medium (Noble Bio, Hwasung, Korea). Nucleic acid extraction was performed using the PowerEXP 32 NA Extractor and the PowerPrep Viral NA Extraction Kit EXP (KogeneBiotech, Seoul, Korea). Briefly, 200 µL of the specimen was used to extract 70 µL of nucleic acid.

### 2.3. Multiplex real-time PCR

The multiplex real-time PCR assay (KogeneBiotech) consisted of the PowerChek Respiratory Virus Panel and the PowerChek Bacterial Pneumonia Real-time PCR Kit series. The PowerChek Respiratory Virus Panel targets 16 respiratory viruses, including SARS-CoV-2, influenza A virus, influenza B virus, enterovirus/rhinovirus, human coronavirus NL63, human coronavirus OC43, human coronavirus 229E, human parainfluenza viruses 1/2/3/4, adenovirus, human metapneumovirus, RSV, and bocavirus. The PowerChek Bacterial Pneumonia Real-time PCR Kit series targets 6 bacterial pathogens: *Streptococcus pneumoniae*, *Legionella pneumophila*, *Haemophilus influenzae*, *M pneumoniae*, *B pertussis*, and *Chlamydophila pneumoniae*.

The PowerChek Respiratory Virus Panel and PowerChek Bacterial Pneumonia Real-time PCR Kit series provide pre-dispensed reverse transcription (RT)-PCR reagents: 10 μL of RT-PCR master mix and 5 μL of the primer/probe mixture in each PCR tube. The set includes human glyceraldehyde-3-phosphate dehydrogenase gene primers and a probe, which serve as an endogenous internal control. Amplification was initiated by loading 5 μL of template ribonucleic acid or deoxyribonucleic acid into each PCR tube. The RT-PCR procedure was conducted using a CFX96 Real-Time PCR Detection System (Bio-Rad Laboratories), with a total turnaround time of 1 hour and 20 minutes, using the following thermal profile: an initial RT step at 50°C for 15 minutes, followed by RT inactivation at 95°C for 5 minutes.

This was followed by 5 preamplification cycles at 95°C for 10 seconds and 60°C for 40 seconds, without fluorescence collection. Finally, 40 amplification cycles were conducted at 95°C for 10 seconds and 60°C for 40 seconds, with fluorescence data collected at 60°C during each cycle. The results were analyzed automatically using Kogene Viewer software version 2.05 (KogeneBiotech). A Ct value of <38 was considered indicative of a positive result for the target and internal control. For SARS-CoV-2, the PowerChek assay targets the nucleocapsid gene (N) and the open reading frame 1ab gene, and the results are deemed positive when both target genes are detected. The assay was conducted according to the manufacturer’s guidelines.

### 2.4. Reconfirmation of SARS-CoV-2 by singleplex PCR

In cases where SARS-CoV-2 was detected by multiplex PCR, confirmation was performed with the singleplex real-time PCR assay using the PowerChek 2019-nCoV Real-time PCR kit (KogeneBiotech).

### 2.5. Data analysis

Detection targets were analyzed using the entire panel, bacterial panel, and viral panel, and the results were categorized as “mono-detected,” “co-detected,” “Bacteria and virus co-detected,” and “not detected.” In the entire panel, “not detected” refers to the absence of both bacterial and viral pathogens in the sample, whereas “co-detected” is defined as the simultaneous detection of 2 or more types of microbes, regardless of whether they are bacteria or viruses. The result category “Bacteria and virus co-detected” was not separately classified in this panel. In the bacterial and viral panels, “not detected” indicates the absence of the respective microbial group (bacteria or viruses), without considering the detection of the other microbial group. In both panels, “co-detected” is defined as the simultaneous detection of 2 or more microbial targets within the same microbial group (bacteria or viruses). Samples with simultaneous detection of both bacteria and viruses were classified as “Bacteria and virus co-detected” in both panels. For the analysis, the chi-square test was applied to compare categorical variables, and the Mann–Whitney *U* test was used for nonparametric comparisons of continuous data using MedCalc version 11.5.1.0 (MedCalc Software, Ostend, Belgium).

## 3. Results

### 3.1. Patient characteristics and type of specimen

During the study period, upper respiratory specimens from 245 pediatric patients (median age: 6 years [IQR 4–10], female ratio: 42.0%) and 373 adult patients (median age: 71 years [IQR 64–81], female ratio: 40.5%) were included. NPSs were the most common specimen type, accounting for 96.3% of pediatric specimens (236/245) and 69.2% of adult specimens (258/373).

### 3.2. Distribution of respiratory bacterial and viral targets

**Figure 1.**
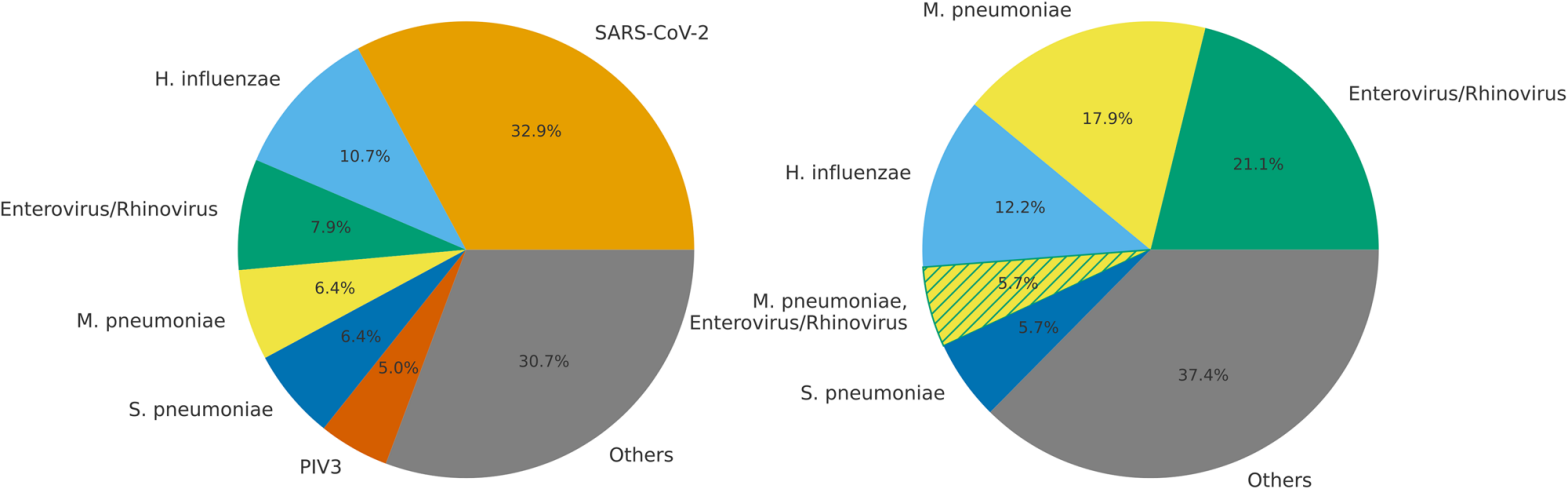
Distribution of respiratory bacterial and viral targets in adults (left) and pediatric patients (right). Results with a frequency of <5% were combined and represented as others. SARS-CoV-2 = severe acute respiratory syndrome coronavirus 2.

In pediatric patients, pathogens were detected in 123 cases. The most frequent result was enterovirus/rhinovirus mono-detection (21.0%, 26/123 detected cases), followed by *M pneumoniae* mono-detection (17.9%, 25 cases) and *H influenzae* mono-detection (12.2%, 15 cases). Co-detection of *M pneumoniae* and enterovirus/rhinovirus was observed in 5.7% of cases (7 cases), and *S pneumoniae* mono-detection was detected at the same frequency (5.7%, 7 cases). In adults, pathogens were detected in 140 cases. SARS-CoV-2 mono-detection was the most frequent result (32.9%, 46/140 detected cases), followed by *H influenzae* mono-detection (10.7%, 15 cases) and *enterovirus/rhinovirus* mono-detection (7.9%, 11 cases). *M pneumoniae* and *S pneumoniae* were also mono-detected in 6.4% of cases each (9 cases each), indicating that the 5 most frequent detection results in adults were all mono-detections. The distribution of respiratory bacterial and viral targets in both age groups is shown in Figure 1.

SARS-CoV-2 was detected in 60 adult patients, regardless of whether it was mono-detected or co-detected. Among adult patients with SARS-CoV-2 detection, the median age was 69 years (IQR 57–82), and 38.8% were female. No statistically significant differences in age or sex distribution were observed between the SARS-CoV-2-detected and SARS-CoV-2-not-detected groups within adult patients (Mann–Whitney *U* test *P* = .79 for age; chi-square test *P* = 1.00 for gender). In pediatric patients, SARS-CoV-2 was detected in 7 cases, of which 4 were mono-detections and 3 were co-detections.

Among the *M pneumoniae*-positive cases, 13 occurred in adults and 40 in children, indicating a significantly higher prevalence in the pediatric population (*P* < .001, chi-square test). The median age of the pediatric patients was 8.0 years (interquartile range [IQR]: 2–14), and the male proportion was 67.5% (27 of 40), indicating a male predominance.

### 3.3. Co-detected cases

In pediatric patients, there were 84 mono-detections and 39 co-detections (5 within the bacterial panel, 7 within the viral panel, and 27 involving bacteria and viruses). In adult patients, there were 105 mono-detections and 35 co-detections (4 within the bacterial panel, 4 within the viral panel, and 27 involving bacteria and viruses). Pediatric patients showed a significantly higher co-detection rate (15.92%, 39/245) than adult patients (9.4%, 35/373; *P* = .02, chi-square test).

**Figure 2.**
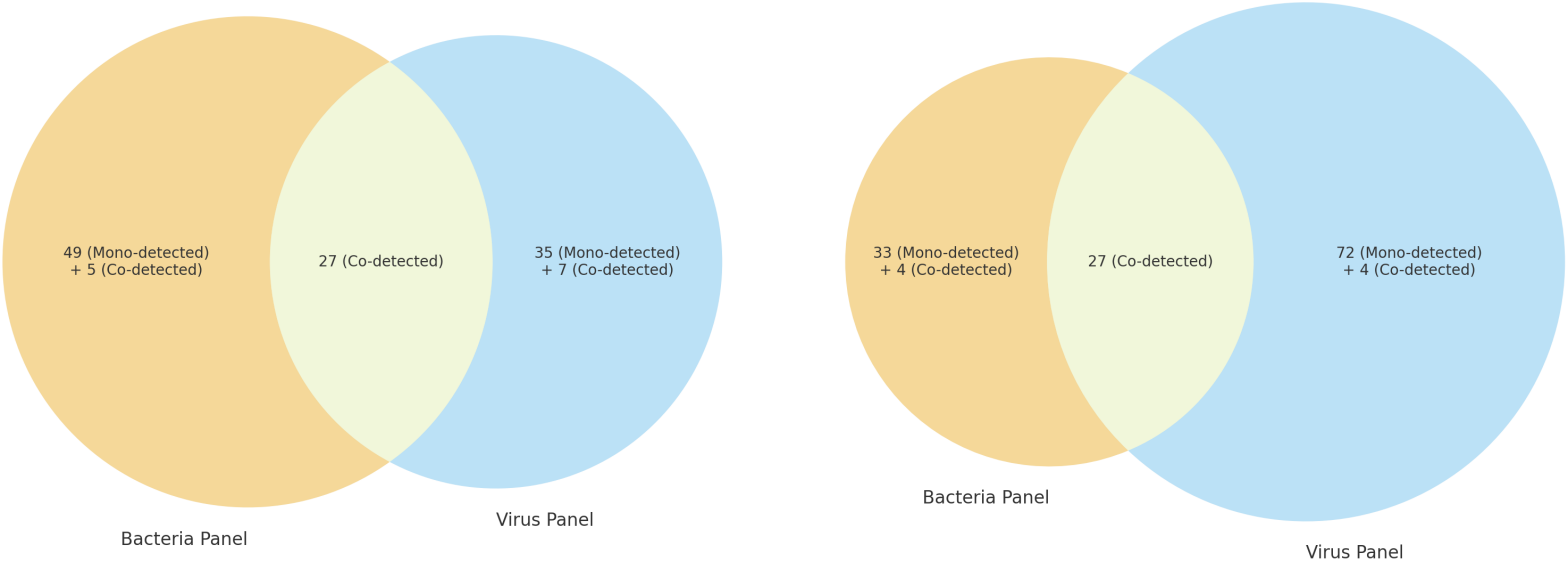
Bacterial and viral targets mono-detected and co-detected in the adult (left) and pediatric (right) patients.

In pediatric patients, the most frequently observed targets among co-detections were enterovirus/rhinovirus (n = 26), *M pneumoniae* (n = 17), and *H influenzae* (n = 12), which are also among the top 3 in the overall pediatric results. In adult patients, the 3 most frequently observed targets within the co-detection results were *S pneumoniae* (n = 18), *H influenzae* (n = 15), and SARS-CoV-2 (n = 14), which were also among the 5 most common targets in the overall results. Among all co-detections, a combination of bacteria and viruses was observed in 27 cases in each group (Fig. 2 and Table 1).

**Table 1:** Respiratory target detections in adult patients and pediatric patients.

	Mono-detected	Co-detected	Bacteria and virus co-detected	Not detected
Adult patients (n = 373)				
Entire panel	105	35		233
Bacteria panel	33	4	27	309
Virus panel	72	4		270
Pediatric patients (n = 245)				
Entire panel	84	39		122
Bacteria panel	49	5	27	164
Virus panel	35	7		176

## 4. Discussion

In the summer of 2024, the Republic of Korea experienced a simultaneous outbreak of multiple respiratory pathogens, including *M pneumoniae*, *B pertussis*, and SARS-CoV-2. This concurrent circulation of diverse pathogens highlighted the clinical challenge of distinguishing among etiologies based on symptoms alone and emphasized the importance of laboratory-based diagnostics for accurate diagnosis and appropriate treatment. The overlapping clinical presentations of these acute respiratory infections necessitated a more comprehensive diagnostic approach. Syndromic panel testing, which allows for the simultaneous detection of multiple pathogens, has emerged as a valuable tool in this context.^[12,13]^ These multiplex assays enable the rapid and concurrent identification of various respiratory pathogens, thereby supporting timely clinical decision-making and facilitating effective infection-control measures.^[12,14]^

Given that the prevalence, distribution, and clinical presentation of respiratory infections may differ between adults and children, as demonstrated in previous studies, including the age-specific analysis by Hwang et al,^[15]^ it is critical to evaluate these differences systematically. Their findings revealed distinct patterns in both mono-infection and co-infection rates according to age group, with higher co-infection rates observed in young children and older adults. Therefore, a detailed comparison of pathogen profiles between pediatric and adult populations during periods of active co-circulation can benefit from syndromic multiplex testing, which comprehensively captures the complexity of infection dynamics across age groups.

This study aimed to compare infection patterns between adult and pediatric patients using a respiratory panel assay capable of detecting 16 viral pathogens and 6 bacterial species. We found that SARS-CoV-2 was detected more frequently in adult patients (60 cases) than in pediatric patients (7 cases), and this difference was statistically significant (*P* < .001). SARS-CoV-2 accounted for the highest frequency among adult mono-detections, suggesting that it continues to cause intermittent, small-scale outbreaks even in the post-pandemic period. The higher detection rate of SARS-CoV-2 in elderly individuals is consistent with previous studies.^[16–18]^ It has also been reported that older adults are more likely to develop severe illness following SARS-CoV-2 infection.^[17,18]^ These findings suggest that, in individuals aged 60 and above, SARS-CoV-2 testing should be prioritized in cases of acute respiratory illness to enable early diagnosis and timely treatment.

In our study, the PowerChek Respiratory Virus Panel assay included SARS-CoV-2 as part of its viral panel. However, most commercially available multiplex respiratory panels do not include SARS-CoV-2 as a target pathogen, as it is generally tested for separately. Therefore, infections may be missed if a separate SARS-CoV-2 test is not intentionally ordered. In fact, among the 67 SARS-CoV-2-positive cases identified in our hospital, only 14 adult patients had undergone SARS-CoV-2 testing for clinical purposes (data not shown). Given the continued detection of SARS-CoV-2, it is important to consider additional testing for SARS-CoV-2 in patients with acute respiratory symptoms, especially those aged 60 years and older, if the virus is not included in the target list of the diagnostic respiratory panel.

Among pediatric patients, enterovirus/rhinovirus and *M pneumoniae* were the most frequently detected pathogens, consistent with previous findings.^[15]^ Notably, human rhinovirus has consistently circulated in pediatric populations throughout both the pandemic and post-pandemic periods. In contrast to other respiratory viruses, rhinoviruses exhibited a high level of resistance to non-pharmaceutical interventions implemented during the pandemic, showing minimal decline even as other viruses markedly decreased in prevalence.^[19]^ This persistent circulation has continued beyond the pandemic, reinforcing the role of rhinovirus as a stable and enduring cause of respiratory infections in children.

In the present study, *M pneumoniae* was frequently detected in pediatric patients, and co-detection of *M pneumoniae* and enterovirus/rhinovirus was one of the most common co-detection patterns in this group. Among *M pneumoniae*-positive cases, pediatric patients represented a larger proportion of cases than adult patients, and most pediatric cases occurred in school-aged children. These findings suggest that *M pneumoniae* was actively circulating among children during the study period and was often detected together with viral respiratory pathogens. Previous studies have reported that *M pneumoniae* infections in children occur more frequently among school-aged children, particularly those aged 5 to 15 years.^[20,21]^ This pattern may be related to age-dependent immune responses as well as increased social interactions and exposure in school settings. *M pneumoniae* infection is also known to show epidemic peaks at intervals of approximately 3 to 7 years.^[21,22]^ In Korea, epidemic peaks were observed in 2015 and 2019, and another epidemic period occurred in 2024, coinciding with the period of the present study.

In addition, previous reports have suggested that co-detection of *M pneumoniae* with viral respiratory pathogens may be clinically relevant.^[23,24]^ However, the present study was designed to evaluate pathogen detection and co-detection patterns using multiplex PCR, and detailed clinical correlations were unavailable. Therefore, our findings should be interpreted as epidemiological data on respiratory pathogen distribution rather than as direct evidence of the clinical consequences of co-detected pathogens. Further studies incorporating clinical characteristics, treatment information, and follow-up data are needed to clarify the clinical significance of these co-detection patterns.

According to previous studies,^[15,25]^ pediatric patients are more likely to experience viral co-infections than adults, and a similar trend was observed in our study. Previous reports have proposed several possible explanations for this phenomenon. Differences in infection patterns between children and adults may reflect age-related physiological and immunological factors. Young children are more susceptible to multiple infections because of their immature immune systems and frequent exposure in communal settings, such as daycare or kindergarten.^[15]^ In contrast, older adults may have weakened immune responses associated with aging, which increases their vulnerability to severe infections.^[25]^ Furthermore, environmental factors such as air pollution may disproportionately affect children because of their developing lungs and higher ventilation rates, whereas underlying comorbidities in the elderly contribute to the increased severity of infections.^[26,27]^

The co-infection rate among SARS-CoV-2-positive patients was higher than that observed among patients positive for other pathogens. Specifically, 20.7% of SARS-CoV-2-positive patients exhibited co-infection with at least one additional respiratory pathogen, which is comparable to previously reported findings.^[28]^ Several studies have reported the clinical implications of SARS-CoV-2 co-infection. A meta-analysis of studies published during the pandemic reported that SARS-CoV-2 co-infection or superinfection was associated with poor clinical outcomes.^[29]^ In a study utilizing K18-hACE2 transgenic mice, co-infection with influenza virus and SARS-CoV-2 was reported to result in a prolonged infection period compared with single infections, along with reduced neutralizing antibody titers and CD4+ T-cell responses against each virus.^[30]^ These clinical and experimental data underscore the need for further research into the co-detection of respiratory pathogens.

This study has several limitations. First, this was a single-center study conducted at 1 tertiary-care hospital, which may limit the generalizability of the findings to other institutions, geographic regions, or healthcare settings with different patient populations and respiratory pathogen epidemiology. Second, the study was conducted over a relatively short 2-month period and, therefore, may not fully capture seasonal variation or long-term trends in respiratory pathogen circulation. Third, detailed clinical outcome data, such as disease severity, treatment, hospitalization course, and prognosis, were not available. As a result, the clinical significance of pathogen detection and co-detection could not be fully assessed. Fourth, because detailed clinical correlations were unavailable, this study could not distinguish active infection from colonization. This is particularly relevant for bacteria such as *H influenzae* and *S pneumoniae*, which may asymptomatically colonize the upper respiratory tract. Fifth, multivariable analyses were not performed because data on potential confounding variables, including healthcare-seeking behavior, testing practices, vaccination status, and underlying comorbidities, were not available in the retrospective dataset. These factors may vary by age group and may influence the likelihood of hospital visits, multiplex PCR testing, pathogen detection, and clinical presentation. Therefore, the observed differences in detection patterns between pediatric and adult patients should be interpreted with caution, and further multicenter studies with longer surveillance periods, detailed clinical information, and multivariable analyses are needed to validate and extend our findings.

Nevertheless, this study provides valuable epidemiological baseline data that may support the diagnosis of acute respiratory infections. By comparing respiratory pathogen detection patterns between adults and children during a period of concurrent pathogen circulation, this study offers insights into the differential epidemiology and co-infection trends across age groups. Future studies with extended surveillance periods, larger sample sizes, and correlations with clinical outcomes are warranted to better understand the impact of co-infections and to guide age-specific diagnostic and therapeutic strategies.

In summary, there is a significant gap in comparative data regarding pediatric and adult cohorts in the aftermath of the SARS-CoV-2 pandemic. Further investigations into the epidemiology of respiratory infections in the post-pandemic era are needed, with particular emphasis on delineating age-specific variations and pathogen-pathogen interactions. Such insights may help optimize diagnostic, preventive, and therapeutic strategies for managing respiratory infectious diseases.

## Author contributions

**Conceptualization:** Soo Young Yoon, Chae Seung Lim, Min-Chul Cho.

**Methodology:** Woong Sik Jang, Seoyeon Park.

**Data curation:** Young Jin Kim, Imseok Kang.

**Writing – original draft:** Young Jin Kim, Min-Chul Cho.

**Writing – review & editing:** Young Jin Kim, Min-Chul Cho.
